# Biological Behavioral Alterations of the Post-neural Differentiated Dental Pulp Stem Cells Through an *in situ* Microenvironment

**DOI:** 10.3389/fcell.2020.625151

**Published:** 2020-12-03

**Authors:** Lihua Luo, Xiaoyan Wang, Yanni Zhang, Yuwei Wu, Fengting Hu, Zhenjie Xing, Lei Wang, Jian Xiao, Fernando Guastaldi, Yan He, Qingsong Ye

**Affiliations:** ^1^School and Hospital of Stomatology, Wenzhou Medical University, Wenzhou, China; ^2^Wenzhou Institute of Biomaterials and Engineering, Wenzhou Medical University, Wenzhou, China; ^3^School of Pharmaceutical Sciences, Wenzhou Medical University, Wenzhou, China; ^4^Skeletal Biology Research Center, Massachusetts General Hospital, Harvard University, Boston, MA, United States; ^5^Tianyou Hospital, Wuhan University of Science and Technology, Wuhan, China; ^6^Center of Regenerative Medicine, Renmin Hospital of Wuhan University, Wuhan, China

**Keywords:** central nervous system, dental pulp stem cells, post-neural differentiation, regenerative medicine, culture microenvironment

## Abstract

Transplantation of undifferentiated dental pulp stem cells (DPSCs) may suffer from tumorigenesis. Neuronal differentiated DPSCs (d-DPSCs) have emerged as an ideal source to treat central nervous system (CNS) disorders. Moreover, different components of culture medium functioned on the characteristics of d-DPSCs *in vitro*. In this study, d-DPSCs were cultured in three types of medium: Neurobasal^®®^-A medium supplemented with 2% B27 (the 2% B27 NM group), Neurobasal^®^ -A medium supplemented with 2% B27 and 5% FBS (the 2% B27 + 5% FBS NM group), and α-MEM containing 10% FBS (the 10% FBS α-MEM group). We found that d-DPSCs in the 2% B27 + 5% FBS NM group had lower proliferation and reduced expression of transient receptor potential canonical 1 (TRPC1) and CD146, whereas up-regulated Nestin and microtubule-associated protein-2 (MAP-2). Notably, d-DPSCs in the 10% FBS α-MEM group possessed high proliferative capacity, decreased expression of neuron-like markers and partially restored stemness. It was demonstrated that d-DPSCs cultured in the 2% B27 + 5% FBS NM could maintain their neuron-like characteristics. Besides, d-DPSCs cultivated in the 10% FBS α-MEM could partially recover their stem cells properties, indicating that neural differentiation of DPSCs was reversible and could open novel avenues for exploring the pluripotency of DPSCs.

## Introduction

The central nervous system (CNS) is the center of all motor output and sensory perception. Neuroinflammation, traumatic events, neurodegenerative disorders, and so on, can cause CNS tissue destruction along with function disablement, severely impacting patients and their families (Hong et al., 2016; Kahveci et al., 2017; Baumard et al., 2018). In the lesion site, apart from the lack of neuronal precursors, activated astrocytes and microglia cells would induce apoptosis of neurons and inhibit regeneration of axons (de Amorim et al., 2020). Recently, stem cells-based therapy has provided a promising treatment option for neuronal injury and disease due to the capacity of stem cells to differentiate into multiple neurogenic lineages (Bianco et al., 2016; Caseiro et al., 2016).

Dental pulp stem cells (DPSCs), which are a type of mesenchymal stem cells (MSCs), have MSCs-like properties such as the ability to undergo self-renewal and multi-differentiation (Gronthos et al., 2000, 2002). DPSCs express MSCs-like phenotypic markers, including CD73, CD90, CD105, CD146, and STRO-1, and do not express hematopoietic lineage surface molecules, such as CD14, CD34, CD45, and HLA-DR (Gronthos et al., 2002; Kawashima, 2012). DPSCs, which originate from embryonic neural crest ecto-MSCs and oral-derived epithelial stem cells, can be readily isolated from extracted human teeth such as impacted third molars and orthodontically extracted premolars (Karaoz et al., 2011; Pisciotta et al., 2020). Therefore, DPSCs can be collected without the need for invasive surgical procedures and without ethical concerns (Geng et al., 2017; Xiao et al., 2017). Moreover, DPSCs have the ability to express neural stem cell-like markers such as Nestin, glial fibrillary acidic protein (GFAP), β-III tubulin, and microtubule-associated protein-2 (MAP-2) without the pre-induction of neuronal differentiation (Sakai et al., 2012; Feng et al., 2013). Additionally, upon induction, DPSCs can differentiate into mature neurons, dopaminergic-like cells, Schwann cells, oligodendrocyte progenitors, neuroglial cells, and retinal ganglion-like cells using neurogenic supplements (Heng et al., 2016; Mead et al., 2017; Sanen et al., 2017). Recent reports have shown that DPSCs have a much better neurogenic differentiation capability than MSCs, which are derived from other tissues such as bone marrow and adipose tissue (Luo et al., 2018b). Thus, DPSCs have been applied widely in stem cell therapy to treat CNS diseases such as spinal cord injury, stroke, Parkinson’s disease, Alzheimer’s disease, and retinal degeneration (Martens et al., 2013; Chalisserry et al., 2017; Mead et al., 2017).

Although most previous studies have demonstrated that the transplantation of undifferentiated DPSCs has neuro-regenerative potential for neural lesions in animal models *in vivo*, there are some disadvantages in utilizing undifferentiated DPSCs for neural regeneration (Heng et al., 2016). Firstly, undifferentiated DPSCs have a tendency to undergo spontaneous differentiation into multiple lineages upon grafting, which may attenuate their clinical efficacy because only a small proportion of the transplanted DPSCs differentiate into neural lineages *in vivo*. Secondly, there is a risk that undifferentiated DPSCs can generate undesired lineages at the transplantation site, such as fibrotic scar tissue, which may hinder neural regeneration. Moreover, the injured site of neurological tissue presents an obviously different environment compared with the carefully controlled environment *in vitro*. There are also limitations in that the migration of transplanted DPSCs to the injured site may be limited *in vivo* (Mead et al., 2017). Additionally, as dental pulp derived-MSCs, DPSCs have the potential to undergo tumorigenesis or tumor growth and progression at the transplantation site (Ohkoshi et al., 2017; Ridge et al., 2017). Accordingly, pre-neuronal differentiated DPSCs (d-DPSCs) can express neuron-associated surface molecules before grafting *in vivo*, which may enhance their integration into the host’s nervous system. Meanwhile, the transplantation of pre-neuronal d-DPSCs can also reduce tumorigenesis and promote neurogenesis.

Recently, it has been confirmed that DPSCs can be induced to form neurospheres or differentiate into mature neurons by endogenous signaling cues (Heng et al., 2016). Many factors, for instance, the extracellular matrix, mechanical force, growth factors, culture systems, oxygen stress, and microfluidic systems, have been reported as critical factors for the induction of neuronal differentiation (Heng et al., 2016; Thompson and Chan, 2016; Yamazaki et al., 2016; Kim et al., 2018; Liu et al., 2020). However, few in-depth studies have examined the effects of the surrounding microenvironment on the survival, proliferation, and expression of markers of DPSC post-neuronal differentiation. In this study, we aimed to evaluate the characteristics of post-neuronal d-DPSCs using different *in vitro* culture medium protocols in order to explore the possible development of transplanted neuronal d-DPSCs *in vivo* during stem cell therapy for CNS diseases.

## Materials and Methods

### Primary Isolation and Culture

The isolation and culture procedures for DPSCs were described previously (Luo et al., 2018a), and ethic approval was obtained from the Ethics Committee of Wenzhou Medical University (Project No. 2018008). Briefly, impacted third molars were collected from the Department of Oral and Maxillofacial Surgery, Stomatological Hospital of Wenzhou Medical University, Wenzhou, China. Dental pulp tissues were extracted, rinsed three times in phosphate-buffered saline (PBS), and cut into small pieces. The tissue pieces were treated with 3 mg/mL collagenase type I (Gibco, Gaithersburg, MD) and 4 mg/mL dispase (Sigma-Aldrich, Steinheim, Germany) in a shaker (150–180 rpm) for 10 min at 37°C. Digestion was terminated with 1 mL fetal bovine serum (FBS; Gibco) followed by centrifugation at room temperature for 10 min at 1,500 rpm. The collected cells and pulp tissue were resuspended in α-modified Eagle’s medium (α-MEM; Gibco) supplemented with 20% FBS, 100 U/mL penicillin, and 100 μg/mL streptomycin (Gibco). The cell suspension was plated in cell culture flasks and incubated at 37°C in a 5% CO_2_ humidified atmosphere. The culture medium was initially replaced at day 5, and then replaced every other day. The morphology of DPSCs was observed by light microscopy (TS100; Nikon, Tokyo, Japan), and the proliferation rate of DPSCs was evaluated by a Cell Counting Kit-8 (CCK-8) assay (Dojindo Molecular Technologies, Kumamoto, Japan) as described previously (Luo et al., 2018a).

### Immunophenotype and Multi-Differentiation of DPSCs

The immunophenotype of DPSCs were characterized by immunofluorescence analysis. Briefly, DPSCs were seeded onto glass slides that were placed in 6-well plates, fixed with 4% paraformaldehyde for 30 min, washed three times with PBS, and blocked with 5% bovine serum albumin (BSA; Amresco, Solon, OH, United States) containing 0.1% Triton X-100 (Solarbio, Beijing, China) for 30 min. DPSCs were incubated with primary antibodies against CD146 (1:200; Abcam, Cambridge, United Kingdom) and STRO-1 (1:50; Santa Cruz Biotechnology, Santa Cruz, CA, United States) at 4°C overnight, washed 3 times with Tween-20, and stained with secondary antibodies to donkey anti-rabbit IgG H&L (1:500; Abcam) and donkey anti-mouse IgG H&L (1:500; Abcam) for 1 h at room temperature in the dark. Cell nuclei were stained with 4′,6-diamidino-2-phenylindole (DAPI; Beyotime, Shanghai, China) for 5 min. The glass slides were treated with Antifade Mounting Medium (Beyotime) and observed by fluorescence microscopy (Eclipse 80i; Nikon, Japan).

As for multi-differentiation, DPSCs were cultured to be confluent for osteogenic and adipogenic differentiation. The culture medium was replaced by the OriCell^TM^ osteogenic differentiation medium and adipogenic differentiation medium (all from Cyagen Biosciences, CA, United States), respectively. After being induced for 21 days, cells were fixed in 4% formaldehyde, mineralized nodules and lipid droplets were stained by Alizarin Red solution and Oil Red O solution (all from Sigma-Aldrich), respectively. Concerning chondrogenic differentiation, 2.5 × 10^5^ cells were centrifuged at 1,000 rpm for 5 min at room temperature in a 15 mL conical tube. Afterward, the cell pellets were cultivated in the OriCell^TM^ chondrogenic differentiation medium for 30 days, then this spheroid-like structure was fixed with 4% formaldehyde and embedded in paraffin for Alcian blue staining. Images were taken and analyzed by light microscopy (TS100; Nikon).

### Neurogenic Differentiation of DPSCs and Culture of d-DPSCs

The neurogenic differentiation of DPSCs was described previously (Heng et al., 2016). Briefly, DPSCs were plated in 6-well plates at a density of 1.0 × 10^5^ cells/well in complete α-MEM containing 10% FBS and incubated at 37°C in a 5% CO_2_ humidified atmosphere. After 24 h, the cell culture medium was changed to neuron-inducing Neurobasal^®^-A medium (Thermo Fisher Scientific, Waltham, MA, United States) supplemented with 2% B27 (Thermo Fisher Scientific), 20 ng/mL basic fibroblast growth factor (bFGF; Thermo Fisher Scientific), and 20 ng/mL epidermal growth factor (EGF; Thermo Fisher Scientific) for 12 days. The neuron-inducing medium was replaced every 3 days. On day 12, the cells were determined and verified by immunofluorescence staining with an anti-GFAP antibody (1:200; Sigma-Aldrich).

After 12 days, d-DPSCs were digested, collected, and re-suspended in complete α-MEM with 10% FBS. d-DPSCs were plated in 6-well plates at a density of 1.5 × 10^5^ cells/well. After 24 h, d-DPSCs were divided into 3 different medium groups: Neurobasal^®^-A medium supplemented with 2% B27 (the 2% B27 NM group), Neurobasal^®^-A medium supplemented with 2% B27 and 5% FBS (the 2% B27 + 5% FBS NM group), and complete α-MEM containing 10% FBS (the 10% FBS α-MEM group). Undifferentiated DPSCs that were cultured with complete α-MEM containing 10% FBS were used as the control group. The morphology of the cells was observed by light microscopy (TS100; Nikon).

### CCK-8 Assay

The proliferation of d-DPSCs cultured in the different media was evaluated by a CCK-8 assay (Dojindo Molecular Technologies) as described previously (Luo et al., 2018a). Briefly, d-DPSCs were plated in 96-well plates at a density of 2.0 × 10^3^ cells/well. After 24 h, the cell culture medium was changed to 2% B27 NM, 2% B27 + 5% FBS NM, or 10% FBS α-MEM. Undifferentiated DPSCs treated with complete α-MEM containing 10% FBS, 100 U/mL penicillin, and 100 μg/mL streptomycin were used as the control group. The cell culture medium was changed every other day. After incubation for 1, 3, 6, and 9 days, 10 μL CCK-8 solution was added to 100 μL culture medium of each well and incubated for 1 h at 37°C in a 5% CO_2_ humidified atmosphere. Optical density (OD) was measured photometrically at 450 nm using an absorbance microplate reader (Varioskan LUX; Thermo Fisher Scientific).

### Immunofluorescence Staining

The expression of proliferation (e.g., transient receptor potential canonical 1, TRPC1), stemness (e.g., CD146), and neuronal differentiation (e.g., Nestin and MAP-2) markers was determined in d-DPSCs cultured in the different media by immunofluorescence staining as described previously (Luo et al., 2018a). At the designated time points after culture, the cells were fixed in 4% paraformaldehyde for 30 min and incubated for 30 min in a blocking solution containing 5% BSA (Amresco) and 0.1% Triton X-100 (Solarbio) at room temperature. The cells were incubated with the following primary antibodies at 4°C overnight: anti-TRPC1 (1:50; Santa Cruz Biotechnology), anti-CD146 (1:200; Abcam), anti-Nestin (1:1000; Sigma-Aldrich), and anti-MAP-2 (1:500, Sigma-Aldrich). The secondary antibodies were donkey anti-rabbit IgG H&L and donkey anti-mouse IgG H&L (1:500; Abcam). Cell nuclei were stained with DAPI (Beyotime), and images were taken by fluorescence microscopy (Eclipse 80i; Nikon, Japan). Both the culture of undifferentiated DPSCs in complete α-MEM containing 10% FBS for 6 days and the culture of DPSCs in continuous neuronal differentiation medium for 12 days (the neural-induction group) served as control groups.

### Western Blot Analysis

The protein expression of d-DPSCs cultured in the different media was analyzed by western blotting (Luo et al., 2018a). d-DPSCs were plated in 6-well plates and incubated with 2% B27 NM, 2% B27 + 5% FBS NM, or 10% FBS α-MEM for 6 days. The cells were lysed in sodium dodecyl sulfate (SDS) lysis buffer, and the proteins were collected for western blot analysis. Culture of undifferentiated DPSCs for 6 days and the 12-day neural-induction group were used as control groups. Proteins (20 μg) were separated by 10% SDS-polyacrylamide gel electrophoresis, followed by transfer to a polyvinylidene fluoride membrane (Millipore, Darmstadt, Germany) in transfer buffer. The membranes were blocked with 5% (w/v) milk (BD Bioscience, Franklin Lakes, NJ, United States) in Tris-buffered saline with Tween-20 for 90 min and incubated with the following primary antibodies at 4°C for 16 h: anti-CD146 (1:1000; Abcam), anti-TRPC1 (1:300; Santa Cruz Biotechnology), anti-Nestin (1:1000; Sigma-Aldrich), and anti-MAP-2 (1:300; Santa Cruz Biotechnology). The secondary antibodies were horseradish peroxidase-conjugated and were added to the membranes for 1 h at room temperature. Detection of the target proteins were performed using the ChemiDoc XRS + Imaging System (Bio-Rad Laboratories, Hercules, CA, United States). All experiments were repeated three times.

### Flow Cytometry

The MSCs-like characteristics of d-DPSCs cultured in 10% FBS α-MEM were identified by flow cytometry as described previously (Luo et al., 2018a). Undifferentiated DPSCs cultured in complete α-MEM containing 10% FBS were used as the control group. Briefly, after the cells reached 90% confluence, they were identified by flow cytometry using primary antibodies against human CD90, CD73, CD146, CD105, CD14, CD34, CD45, and HLA-DR (all from BioLegend, San Diego, CA, United States) according to standard protocols. The data were analyzed with a CytoFLEX flow cytometer (Beckman Coulter, Brea, CA, United States).

## Statistical Analysis

Statistical analyses were performed using SPSS 19.0 software (SPSS Inc., Chicago, IL). All data were presented as mean ± standard deviation (SD). One-way ANOVA was used for multiple comparisons. Tukey’s test or Dunnett’s test was used as *post hoc* test. *P* < 0.05 was considered statistically significant.

## Results

### Characterization and Analysis of the Multipotency of DPSCs

**FIGURE 1:**
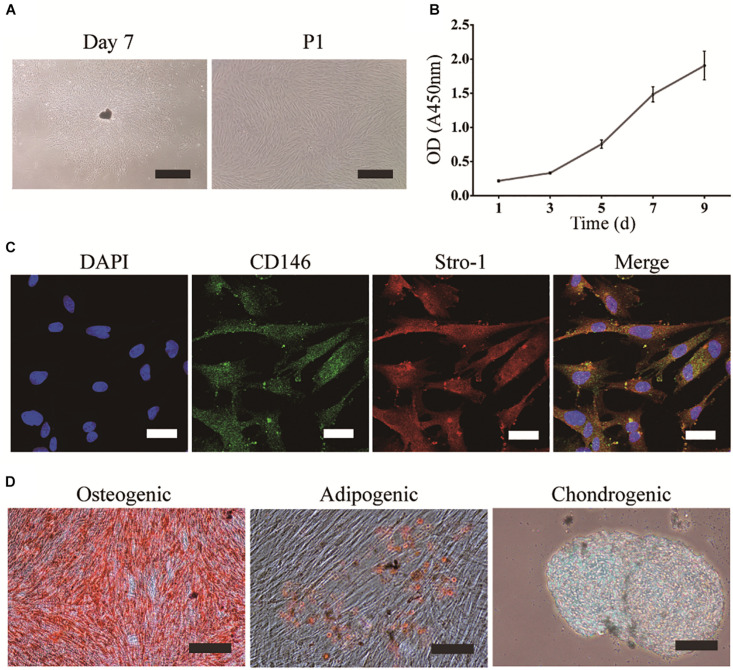
Isolation, culture, and identification of DPSCs. **(A)** DPSC culture at day 7 (scale bar: 500 μm), and the first passage (P1) (scale bar: 200 μm). **(B)** Proliferation of DPSCs at days 1, 3, 5, 7, and 9. **(C)** Expression of CD146 and STRO-1 (scale bar: 33.3 μm). **(D)** Osteogenic, adipogenic, and chondrogenic differentiation of DPSCs (scale bar: 100 μm).

DPSCs were grown in a homogeneous layer of plate-adherent cells and presented with typical fibroblastic and spindle-shaped morphology. DPSCs had a high proliferation rate as OD values (450 nm) increased from 0.2 on day 1 to 1.9 on day 9 (Figures 1A,B). To evaluate the MSCs-like properties of DPSCs, immunocytochemistry and multilineage differentiation were performed. Immunofluorescence staining indicated that DPSCs expressed MSCs-like markers such as CD146 and STRO-1 (Figure 1C). The multipotency of DPSCs was examined by osteogenic, adipogenic, and chondrogenic differentiation. Alizarin Red and Oil Red O staining displayed the deposition of mineralized nodules and formation of lipid droplets, respectively. Moreover, Alcian blue staining indicated that proteoglycans were synthesized during chondrogenic induction (Figure 1D).

### Morphology of Neuronal d-DPSCs and the Effect of Different Culture Media on Their Proliferation

**FIGURE 2:**
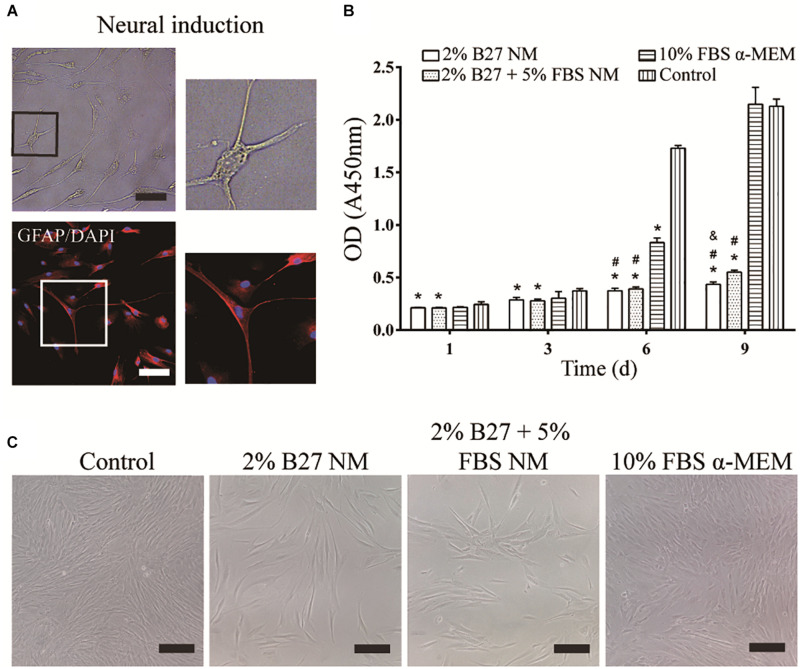
Neurogenic differentiation of DPSCs and their proliferation in the different media. **(A)** d-DPSCs showed a typical neuron-like cell morphology (scale bar: 100 μm). **(B)** Proliferation of d-DPSCs after culture in the different media. **(C)** Morphology of d-DPSCs cultured in the different media at day 6 (scale bar: 200 μm). *P < 0.05 vs. Control group; ^#^*P* < 0.05 vs. 10% FBS α-MEM group; ^&^*P* < 0.05 vs. 2% B27 + 5% FBS NM group.

After neurogenic-induced differentiation with Neurobasal^®^-A medium (Thermo Fisher Scientific) containing 2% B27 (Thermo Fisher Scientific), 20 ng/mL bFGF, and 20 ng/mL EGF (Thermo Fisher Scientific) for 12 days, DPSCs showed neuron-like cellular morphology with a large perikaryon and peripheral cytoplasmic extensions, and expressed the neuronal marker GFAP (Figure 2A). The effect of the different culture media on the proliferation of d-DPSCs was evaluated by a CCK-8 assay (Figure 2B). The results indicated that d-DPSCs grew slowly in the 2% B27 NM and 2% B27 + 5% FBS NM groups, and their viability in these groups was significantly different compared to the control group until day 9 and with the 10% FBS α-MEM group at days 6 and 9. The proliferation of d-DPSCs in the 10% FBS α-MEM group was significantly different compared with the control group at day 6, but was similar to that of the control group at day 9. The morphology of d-DPSCs varied in the different culture media; for example, the cells tended to display a fibroblast-like shape in the 10% FBS α-MEM group at day 6 (Figure 2C). Moreover, cell density in the 10% FBS α-MEM group was similar to that in the control group, but much higher than that of the 2% B27 NM and 2% B27 + 5% FBS NM groups.

### Protein Expression and Immunofluorescence Staining of Proliferation and Stemness Markers of d-DPSCs After Culture in the Different Media

**FIGURE 3:**
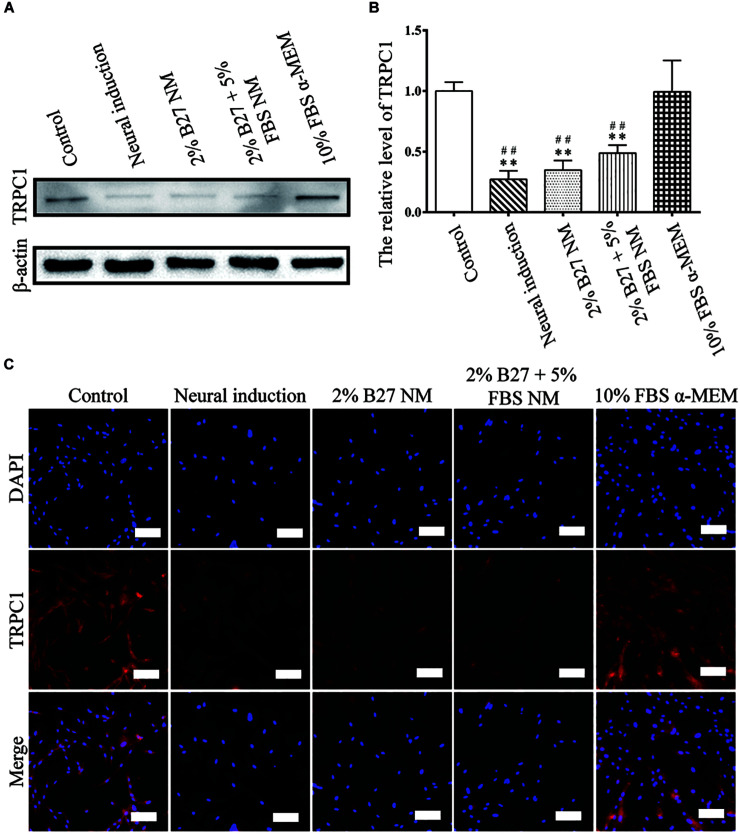
Expression of the proliferation marker TRPC1 in d-DPSCs cultured in the different media. **(A)** Expression of TRPC1 protein. **(B)** Quantification of TRPC1 expression. **(C)** Immunofluorescence staining of TRPC1 (scale bar: 100 μm). **P < 0.01 vs. Control group; ^##^*P* < 0.01 vs. 10% FBS α-MEM group.

The proliferation and stemness characteristics of d-DPSCs in the 2% B27 NM, 2% B27 + 5% FBS NM, and 10% FBS α-MEM groups were verified by western blot and immunofluorescence staining analyses. The results indicated that TRPC1 protein expression was much higher in the 10% FBS α-MEM group than in the other experimental groups and in the neural-induction group, but similar to that of the control group (Figure 3). TRPC1 expression was not significantly different between the 10% FBS α-MEM and control groups (Figure 3B). According to immunofluorescence staining analysis, TRPC1 expression was maintained at a higher level in the 10% FBS α-MEM and control groups compared to the other groups, which was consistent with the data from western blot analysis (Figure 3C).

**FIGURE 4:**
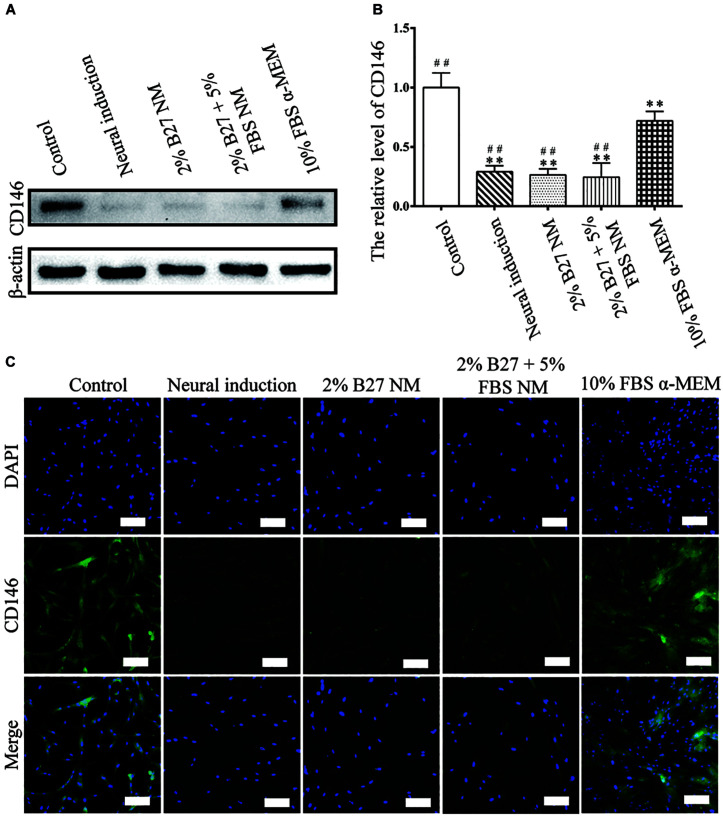
Expression of the stemness marker CD146 in d-DPSCs cultured in the different media. **(A)** Expression of CD146 protein. **(B)** Quantification of CD146 expression. **(C)** Immunofluorescence staining of CD146 (scale bar: 100 μm). **P < 0.01 vs. Control group;^ ##^*P* < 0.01 vs. 10% FBS α-MEM group.

CD146 protein expression was examined to evaluate the stemness properties of d-DPSCs cultured in the different media. In the experimental groups, CD146 expression was the highest in the 10% FBS α-MEM group; however, it was lower than that of the control group. Moreover, CD146 expression was barely detected in the neural-induction group (Figures 4A,B). CD146 was expressed at a high level in the 10% FBS α-MEM and control groups, which was similar to the results from western blot analysis (Figure 4C).

### Protein Expression and Immunofluorescence Staining of Neuronal Markers of d-DPSCs After Culture in the Different Media

**FIGURE 5:**
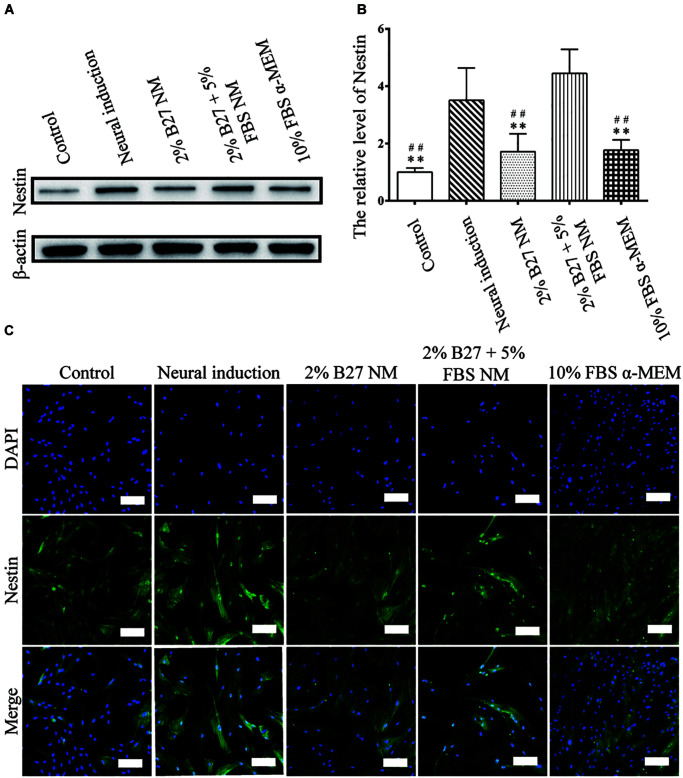
Expression of the neuron-like marker Nestin in d-DPSCs cultured in the different media. **(A)** Expression of Nestin protein. **(B)** Quantification of Nestin expression. **(C)** Immunofluorescence staining of Nestin (scale bar: 100 μm). **P < 0.01 vs. Neural-induction group; ^##^*P* < 0.01 vs. 2% B27 + 5% FBS NM group.

**FIGURE 6:**
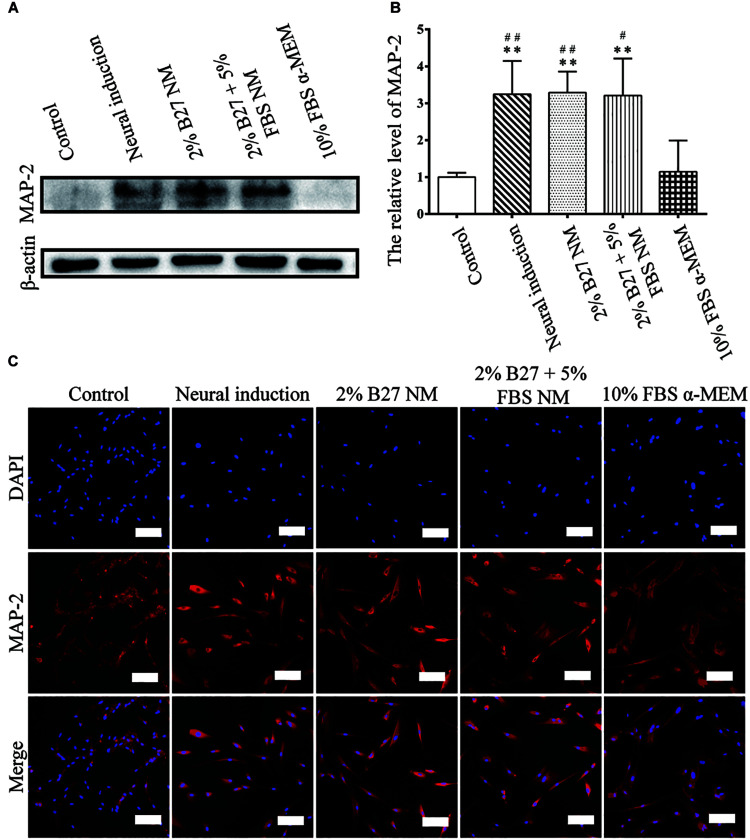
Expression of the neuron-like marker MAP-2 in d-DPSCs cultured in the different media. **(A)** Expression of MAP-2 protein. **(B)** Quantification of MAP-2 expression. **(C)** Immunofluorescence staining of MAP-2 (scale bar: 100 μm). **P < 0.01 vs. Control group; ^##^*P* < 0.01 vs. 10% FBS α-MEM group; ^#^*P* < 0.05 vs. 10% FBS α-MEM group.

The expression of neuronal markers (e.g., Nestin and MAP-2) was examined in d-DPSCs in the 2% B27 NM, 2% B27 + 5% FBS NM, and 10% FBS α-MEM groups. Nestin and MAP-2 expression was higher in the 2% B27 + 5% FBS NM and neural-induction groups. Whereas, Nestin and MAP-2 expression was decreased in the 10% FBS α-MEM and control groups. MAP-2 was expressed at a high level in the 2% B27 NM group, but Nestin was expression at a low level (Figures 5A,B, 6A,B). Immunofluorescence staining indicated that Nestin and MAP-2 were expressed in all experimental and control groups, with significantly increased expression in the 2% B27 NM, 2% B27 + 5% FBS NM, and neural-induction groups (Figures 5C, 6C).

### Expression of MSCs-Like Markers by d-DPSCs in the 10% FBS α-MEM Group

**FIGURE 7:**
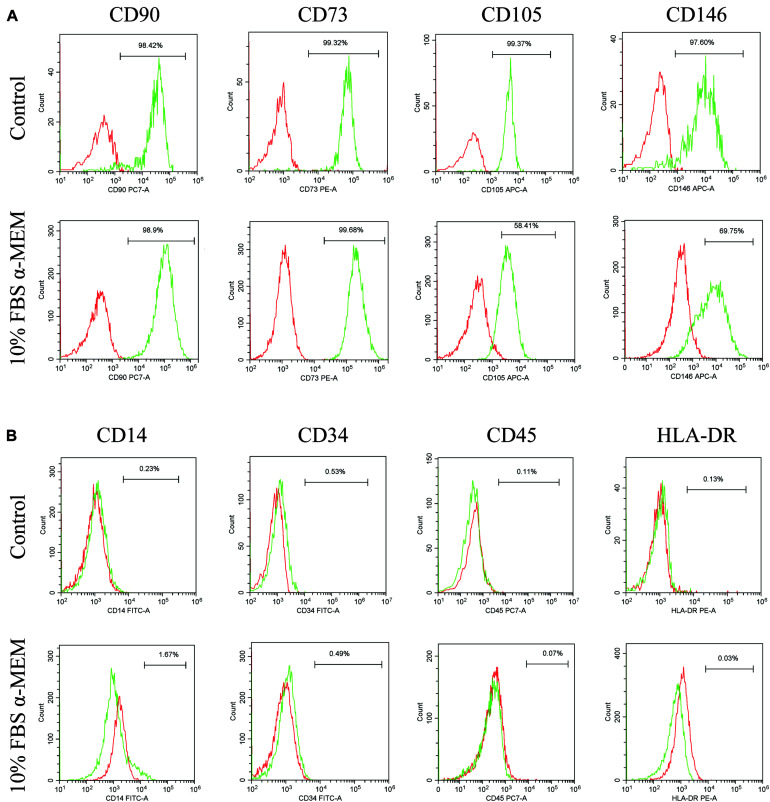
Expression of MSCs-like markers by d-DPSCs in the 10% FBS α-MEM group. **(A)** Positive expression of d-DPSC surface markers. **(B)** Negative expression of d-DPSC surface markers.

According to the results of CCK-8, western blot, and immunofluorescence staining analyses, the characteristics of d-DPSCs in the 10% FBS α-MEM group were similar to those of the control group. Therefore, the MSCs-like properties of d-DPSCs in the 10% FBS α-MEM group were evaluated by flow cytometry using the MSCs markers CD73, CD90, CD105, CD146, CD34, CD14, CD45, and HLA-DR. Flow cytometry demonstrated that d-DPSCs were positive for CD73, CD90, CD105, and CD146 in the 10% FBS α-MEM group, where the expression of CD105 and CD146 was reduced compared to the control group; CD105 and CD146 expression was decreased from 99.37 to 58.41% and from 97.60 to 69.75%, respectively (Figure 7A). d-DPSCs did not express CD34, CD14, CD45, and HLA-DR in the 10% FBS α-MEM group, which was in line with the properties of MSCs in the control group (Figure 7B).

## Discussion

DPSCs, which are derived from the cranial neural crest, have MSCs-like biological properties and possess the capacity to differentiate into neuron-like cells and secrete neuron-related trophic factors (Heng et al., 2016; Kumar et al., 2017). Recently, it has been reported that non-differentiated and d-DPSCs are emerging as new cell sources for the treatment of CNS diseases, such as spinal cord injury, stroke, Parkinson’s disease, and Alzheimer’s disease (Mead et al., 2017). DPSCs have huge advantages over other tissue-derived stem cells for CNS therapy due to their ease of harvesting without invasive surgery, low immunogenicity, and high expression of neuron-like markers (e.g., GFAP, β-III tubulin, MAP-2, and Nestin) without pre-induced differentiation (Sakai et al., 2012; Özdemir et al., 2016). Moreover, DPSCs have vascularization and immunomodulatory properties, which can directly or indirectly stimulate the formation of new blood vessels and enhance blood supply to sites of injury (Nakashima et al., 2009; Gorin et al., 2016; Piva et al., 2017). Thus, DPSCs have been used widely in the field of CNS treatment either by the transplantation of a DPSC suspension alone or in combination with biomaterial scaffolds (Zhang et al., 2016; Mead et al., 2017). However, the microenvironment of transplanted DPSCs in injured neurological tissue is vastly different from that of the induction environment *in vitro* (Mead et al., 2017). Moreover, there is a possible risk of tumorigenesis with DPSCs due to their MSCs biological properties such as multi-differentiation (Ohkoshi et al., 2017; Ridge et al., 2017). Taken together, *in vitro* neuronal d-DPSCs are emerging as a more appropriate cell source for the future treatment of CNS diseases. Therefore, it is important to understand how the neuron-like characteristics of DPSCs can be maintained after differentiation. In this study, we used various types of culture medium to simulate different *in vivo* microenvironments for neuronal d-DPSCs over an extended period of time. We characterized these d-DPSCs using CCK-8, western blot, immunofluorescence staining, and flow cytometry analyses in comparison to undifferentiated DPSCs.

DPSCs are adult stem cells that possess enormous proliferative capacity, whereas neuron-like cells act as terminally differentiated cells with weak proliferative ability (Heng et al., 2016). We hypothesized that the microenvironment of d-DPSCs not only has an impact on their neuron-like characteristics but also restores their stemness. A CCK-8 assay showed that d-DPSCs in the 2% B27 NM and 2% B27 + 5% FBS NM groups grew slowly from days 1 to 9. Whereas, the survival and proliferation of d-DPSCs in the 10% FBS α-MEM group showed a sharp increase from days 6 to 9. The proliferation of d-DPSCs was not significantly different between the 10% FBS α-MEM and control groups at day 9. Moreover, the morphology of d-DPSCs also subsequently changed into a typical MSCs-like phenotype in which fibroblast-like elongation was observed. Further, the effects of the microenvironment on proliferation (e.g., TRPC1), stemness (e.g., CD146), and neuron-like (e.g., Nestin and MAP-2) markers of d-DPSCs supported our hypothesis.

Multiples studies have reported that the biological functions of MSCs, including differentiation, proliferation, and apoptosis, are mediated by intracellular calcium (Ca^2+^) concentration (Deng et al., 2015; Peng et al., 2016). TRPC1 is a voltage-independent membrane channel that mobilizes the extracellular calcium pool to induce Ca^2+^ influx (Clapham, 2003). Torossian et al. (2010) reported that TRPC1 is expressed by MSCs and plays an important role in their proliferation. According to our study, TRPC1 was expressed at the highest level in the 10% FBS α-MEM group, at a similar level to that of the control group. Whereas, TRPC1 was barely expressed in the other experimental groups and the neural-induction group. These results were consistent with those of the CCK-8 assay, which indicated that d-DPSCs cultured in 10% FBS α-MEM had a greater proliferative capacity.

CD146 is an integral membrane glycoprotein that mediates vascular endothelial cell activity and angiogenesis. Notably, CD146 is recognized as an appropriate marker of the stemness of MSCs (Lv et al., 2014). CD146 expression indicates that MSCs possess high clonogenicity and multipotency as well as hematopoiesis capacity (Russell et al., 2010; Lv et al., 2014). As shown in Figure 4, CD146 expression was significantly higher in d-DPSCs of the 10% FBS α-MEM group than in the other experimental groups, but this was not significantly lower than that of the control group, in which undifferentiated DPSCs were cultured. These results indicated that d-DPSCs did lose their stemness during the induction of neural differentiation, while their stemness could be partially preserved using normal MSCs culture medium.

Nestin expression indicates endothelial progenitors and/or newly formed endothelium. Under normal conditions, human dental pulp does not contain true lymphatic vessels, as the lymph drainage system of human dental pulp is poorly developed, and this system consists of interstitial tissue channels devoid of endothelium. In stressed pulp, lymphangiogenesis is characterized by vessel formation. Nesin^+^ cells reportedly participate in angiogenesis as MSCs or endothelial progenitor cells in several tissues and have been found in lymph nodes (Koning et al., 2016). The literature indicates that Nestin expression characterizes a subset of bone marrow perivascular MSCs (Xie et al., 2015). Nestin is a neuronal stem cell marker and its expression is considered a prerequisite for stem cells to differentiate into a neural lineage (Kanao et al., 2017). One study reported that murine DPSCs expressing Nestin at a high level have a considerable capacity to differentiate into a neuron-like phenotype and increase the expression of MAP-2, a marker of mature neurons (Young et al., 2016). MAP-2 associates with actin during early axonal development (Tatiana et al., 2010; Kanao et al., 2017). Our previous studies demonstrated that Nestin expression is significantly increased in DPSCs upon neural-induced differentiation (Luo et al., 2018a). In the present study, Nestin and MAP-2 were highly expressed in d-DPSCs cultured in 2% B27 + 5% FBS NM, similar to the levels observed in the neural-induction group. Therefore, the 2% B27 + 5% FBS NM culture medium could provide an appropriate microenvironment for d-DPSCs to maintain their neuron-like characteristics *in vitro*. Nestin and MAP-2 were expressed at very low levels in d-DPSCs of the 10% FBS α-MEM and control groups, suggesting that the biological features of d-DPSCs in the 10% FBS α-MEM group were very similar to those of undifferentiated DPSCs.

DPSCs maintain the properties of MSCs and express MSCs-like markers, including CD73, CD90, CD105, and CD146, but do not express hematopoietic cell markers, such as CD34, CD14, CD45, and HLA-DR (Gronthos et al., 2002; Kawashima, 2012). In our study, flow cytometric analysis demonstrated that d-DPSCs in the 10% FBS α-MEM group were positive for CD73, CD90, CD105, and CD146, and negative for CD34, CD14, CD45, and HLA-DR. However, CD105 and CD146 expression was lower than that observed in the control group; CD105 and CD146 expression was decreased from 99.37 to 58.41% and from 97.60 to 69.75%, respectively. CD146 and CD105 are both primarily endothelial cell markers associated with vascular endothelial cell activity and angiogenesis (Nakashima et al., 2009; Gao et al., 2020). These results again suggested that d-DPSCs cultured in 10% FBS α-MEM had the ability to restore partly their stemness but had less capacity for angiogenesis.

Recently, stem cells-based therapy has provided a fascinating new approach for the repair of CNS diseases. d-DPSCs have become an attractive source over than the other types of MSCs because of their optimal neurogenic and neurotrophic properties. However, cellular therapy usually requires amplification of cell population. And repetitive passage will ultimately lead d-DPSCs to enter an irreversible proliferation-arrested state and pluripotency-lost position. Therefore, it is very important to provide a suitable method to maintain the biological characteristics of d-DPSCs. Our findings provide clues and cues in modulating the de-differentiation of DPSCs, and it also proves DPSCs as an ideal source for stem cell-based nerve tissue engineering.

## Conclusion

Our study is the first to evaluate the effects of the surrounding microenvironment on the properties of neural d-DPSCs. Our results clearly demonstrated that the basic Neurobasal^®^-A medium supplemented with 2% B27 and 5% FBS could provide a suitable culture microenvironment for d-DPSCs to maintain their neuron-like characteristics *in vitro*. Moreover, d-DPSCs cultured in complete α-MEM containing 10% FBS could partially recover their stem cell properties, indicating that the neural differentiation of DPSCs has a reversible characteristic, which might provide a novel strategy for future research into the multi-differentiation of DPSCs. Furthermore, DPSCs seem to be a promising source of stem cells for nerve regeneration.

## Data Availability Statement

The raw data supporting the conclusions of this article will be made available by the authors, without undue reservation.

## Author Contributions

QY and YH: conceptualization and supervision. LL, XW, YZ, YW, FH, ZX, and LW: methodology and validation. XW, YZ, and LL: data curation and analysis. LL and XW: writing-original draft preparation. JX, FG, and YH: writing-review and editing. YH and QY: funding acquisition. All authors have read and agreed to the published version of the manuscript.

## Conflict of Interest

The authors declare that the research was conducted in the absence of any commercial or financial relationships that could be construed as a potential conflict of interest.
